# Different methods of cell quantification can lead to different results: a comparison of digital methods using a pilot study of dendritic cells in HIV-positive patients

**DOI:** 10.4317/medoral.23472

**Published:** 2020-03-06

**Authors:** Diego Tetzner Fernandes, Willie F.P. van Heerden, Ana Carolina Prado Ribeiro, Thaís Bianca Brandão, Evandro Sobroza de Mello, Cesar Rivera, Marlene B. van Heerden, Rogerio Gondak, Alan Roger Santos-Silva, Pablo Agustin Vargas, Marcio Ajudarte Lopes

**Affiliations:** 1 DDS, MSc, PhD. Oral Diagnosis Department, Piracicaba Dental School, University of Campinas (UNICAMP), Piracicaba, São Paulo, Brazil; 2 DDS, MSc, PhD. Department of Oral Pathology and Oral Biology, School of Dentistry, University of Pretoria, Pretoria, South Africa; 3 DDS, MSc, PhD. Dental Oncology Service, Instituto do Câncer do Estado de São Paulo, ICESP-FMUSP, São Paulo, Brazil; 4 MD, MSc, PhD. Dental Oncology Service, Instituto do Câncer do Estado de São Paulo, ICESP-FMUSP, São Paulo, Brazil; 5 DDS, MSc, PhD. Oral Pathology and Medicine Research Group, Department of Basic Biomedical Sciences, Faculty of Health Sciences, Universidad de Talca, Chile; 6 DDS, MSc, PhD. Department of Pathology, Federal University of Santa Catarina, Florianopólis, Santa Catarina, Brazil

## Abstract

**Background:**

Although new digital pathology tools have improved the positive cell quantification, there is a heterogeneity of the quantification methods in the literature. The aim of this study was to evaluate and propose a novel dendritic cells quantification method in squamous cell carcinoma comparing it with a conventional quantification method.

**Material and Methods:**

Twenty-six squamous cell carcinomas HIV-positive cases affecting the oropharynx, lips and oral cavity were selected. Immunohistochemistry for CD1a, CD83, and CD207 was performed. The immunohistochemical stains were evaluated by automated examination using a positive pixel count algorithm. A conventional quantification method (unspecific area method; UA) and a novel method (specific area method; SA) were performed obtaining the corresponding density of positive dendritic cells for the intratumoral and peritumoral regions. The Mann-Whitney U test was used to verify the influence of the quantification methods on the positive cell counting according to the evaluated regions. Data were subjected to the ANOVA and Student’s t-test to verify the influence of the tumour location, stage, histological grade, and amount of inflammation on the dendritic cells density counting.

**Results:**

The cell quantification method affected the dendritic cells counting independently of the evaluated region (*P*-value <0.05). Significant differences between methods were also observed according to the tumour features evaluations.

**Conclusions:**

The positive cell quantification method influences the dendritic cells density results. Unlike the conventional method (UA method), the novel SA method avoids non-target areas included in the hotspots improving the reliability and reproducibility of the density cell quantification.

** Key words:**Cell counts, immunohistochemistry, dendritic cells, HIV, head and neck neoplasms.

## Introduction

The implementation of combined antiretroviral therapy (cART) has increased the life expectancy of HIV-positive patients. However, new clinical challenges are being associated with HIV-positive patients such as the development of non-AIDS defining malignancies (1-6). Therefore, the incidence of head and neck cancer (HNC) has markedly increased since the widespread use of cART (1,7-11). During the multistep carcinogenesis events, changes in host immunological factors have been observed; thus, studying these complex interactions is crucial for a better understanding of such malignancies (12).

Dendritic cells (DCs) have a central role in the regulation of immunological responses, including antitumour immunity. DCs constitute a heterogeneous population of cells, where the immature cells have high phagocytic activity and the mature cells have high cytokine-producing capacity, conditions that maintain a balance between innate and adaptive immunity (13). The major DCs population in the mucosa epithelium are the Langerhans cells (LCs). LCs may have important roles in the course of an HIV infection, including the probable initial uptake of HIV transmission to the lymph nodes and subsequent transfer to T cells (14). Furthermore, LCs migration inhibition caused by tumor-derived factors prevents LCs from promoting antitumoral immunity (15-17).

Previous studies have shown the low density of LCs in squamous cell carcinoma (SCC) of the skin (18), uterine cervix (19), and anal mucosa (20). Although the relationship among CD4+ T (T helper cells) cell counts, HIV viral load, and LCs density remains unclear (21), these studies suggest that immunological changes associated with HIV infection are predisposing factors to the development of SCC. Once there are no studies that investigate DCs in HNC in HIV-positive patients, the quantification of these cells is prime for a better understanding of this subject and may reveal important data for future steps. However, a great variability of values has been noticed in studies investigating the density of DCs (19,22-25), which complicate the use of published data as reference for new studies.

New digital pathology tools have improved the positive cell quantification process. The automated examination involving the positive pixel count algorithm is replacing the old manual methods since it allows to explore the association of different digital tools. However, the conventional methods for quantifying specific cells in SCC do not consider the variability of the tumour morphological presentation or the amount of inflammation in the evaluated selected areas. Normally, a 1-mm2 hotspot area of a well or moderately differentiated SCC has a different amount of tumour when compared with a poorly differentiated SCC area. Similar bias can occur in regions with different amount of inflammation, regarding the evaluation of immune cell as DCs. The conventional methods quantify cells in the total hotspot areas, so non-target areas, such as fibrosis, can be wrongly considered. Hence, the reproducibility of the studies is affected.

Thus, the aim of the present study was to evaluate a novel DCs quantification method in SCC, comparing it with a conventional method. The research null hypothesis is that a specific area quantification method does not influence the positive cell counting results.

## Material and Methods

Paraffin-embedded tissue samples from 26 SCCs HIV-positive cases affecting the oropharynx, lips and oral cavity were selected from the Departments of Pathology at Instituto do Câncer do Estado de São Paulo, Brazil, and the University of Pretoria, Pretoria, South Africa. The pathological reports were examined for demographic data. The histological grade was revised by two oral pathologists according to a classification proposed by the World Health Organization (26) while the amount of inflammation was evaluated in the peritumoral areas.

Immunohistochemistry was performed on 3 μm formalin-fixed paraffin-embedded tissue sections using the Ventana Benchmark GX automated system (Ventana Medical Systems Inc., Tucson, Arizona). Epitope retrieval to demonstrate CD1a (T-cell surface glycoprotein CD1a; EP3622), CD83 (CD83 antigen; 1H4b) and CD207 (C-type lectin domain family 4 member K; 12D6) expression was performed in high pH retrieval buffer for 56 min for CD1a and CD83, and in low pH retrieval buffer for 48 min for CD207. The incubation was performed with Cell Marque monoclonal rabbit antihuman RTU CD1a (Cell Marque, CA, USA; no dilution required) for the CD1a, a 1:40 Novocastra monoclonal mouse antihuman CD83 (Leica BioSystems Ltd., Newcastle, UK) for the CD83, and a 1:50 Novocastra monoclonal mouse antihuman Langerin (Leica BioSystems Ltd., Newcastle, UK) for the Langerin. The antibodies were detected with the Ventana OptiView DAB Detection System. Sections were counterstained in Haematoxylin, dehydrated and mounted with permanent mounting media. Positive controls were included in all reactions in accordance with the manufacturer’s protocols. The selection of the antibodies was based on their well-established biological properties and previous investigations in different neoplasms

The immunohistochemical stains were evaluated by automated examination based on a previous methodology (25). All slides were scanned with the Scan Scope Aperio System (Aperio, Vista, CA, USA) obtaining a high-quality resolution digital image. The automated staining intensity was quantified with the IMAGESCOPE software (Aperio) using a membrane positive pixel count algorithm. The staining intensity was classified as strongly positive (red), positive (orange), weakly positive (yellow), or negative (blue). For the proper quantification and to avoid background staining, only the strongly positive results were considered.

**Figure 1:**
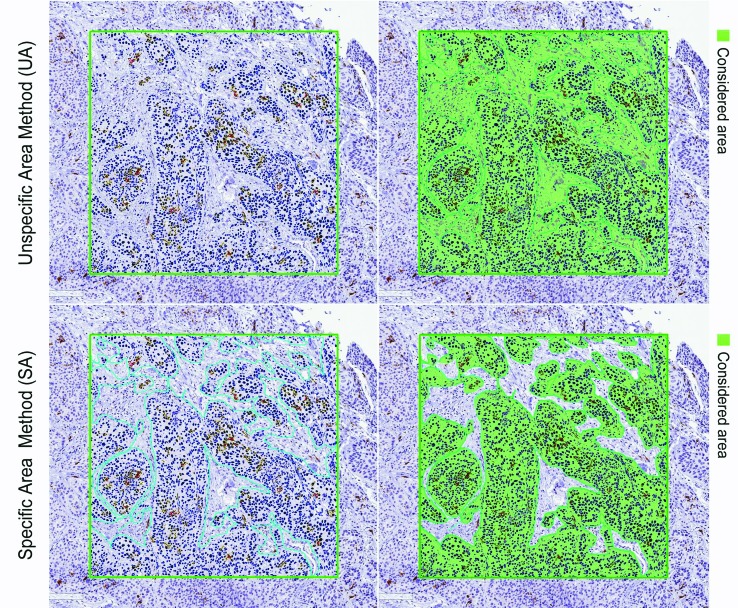
Immunohistochemical detection of CD1a+ dendritic cells in an intratumoral hotspot field using Image Scope software. The green square lines on the left panels represent the 1 mm² hotspot demarcation. The green areas on the right panels represent the considered area for each method (UA: the whole hotspot area; SA: only the tumor area). The software was performed and the strongly positive pixel cells were computed.

For each case, the conventional quantification method (unspecific area method; UA) and the new method (specific area method; SA) were applied. Initially, four 1-mm² hotspots were selected for both intratumoral and peritumoral areas. For the UA method, the positive pixel count software was performed on the total of each 1-mm² hotspot obtaining an average of positive DCs (number of cells/mm²) for each case (average of all hotspots). For the SA method, the exact tumour (intratumoral analyses) or inflammation (peritumoral analyses) areas inside of each 1-mm² hotspot were demarcated and the positive pixel count software was performed only on these specified areas (Fig. 1).

The density of DCs was then calculated using the formula n/DA, where n is the number of positive DCs and DA is the demarcated area (mm²) of each hotspot.

The data analysis was generated using SAS software (The SAS System, 9.4. SAS Institute Inc., Cary, NC, USA, 2012. The normality of the residues was evaluated by the Shapiro-Wilk test and the asymmetry and kurtosis coefficients. Parametric techniques were adopted for variables adherent to Gaussian distribution and nonparametric techniques when the adherence was not satisfactory. The Mann-Whitney U test was used to verify the influence of the quantification methods on the positive cell counting according to the evaluated regions (total, intratumoral or peritumoral). Then, mixed linear generalized models of analysis of variance (ANOVA) were adjusted to test the effects of tumour location, tumour stage, histological grade, and amount of inflammation on positive cell counting. Multiple comparisons of averages based on Student’s t-test was done. The significance level was fixed at 5% for all statistical tests.

## Results

A total of 26 cases of HIV-positive patients were selected. The summary of the clinicopathological data is presented in Table 1.

**Figure 2:**
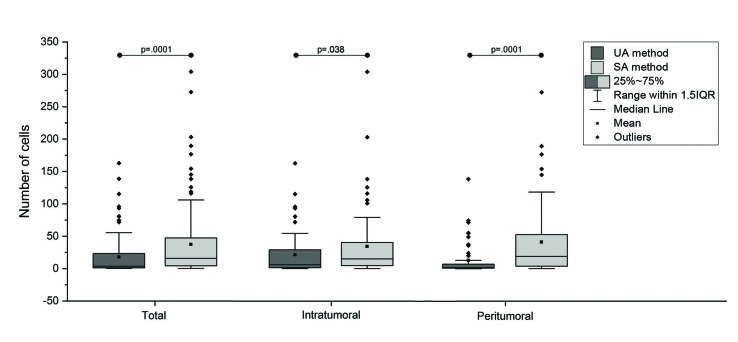
Positive cell counting distribution according to the quantification method (UA or SA) and the evaluated region (total, intratumoral, and peritumoral). Groups connected by lines presents differences between them (Mann-Whitney U test, *P*<0.05).

A total of 624 positive pixel count analyses were performed. All the proteins markers tested (CD1a, CD83, and CD207) were present in the DCs membranes of the SCC samples. The average of the demarcated areas (DA) for the SA method group analyses was 0.60 mm² ±0.23 mm² (mean ±standard deviation) for the intratumoral regions, and 0.21 mm² ±0.13 mm² for the peritumoral regions analyses. The quantification method affected the positive cells counting independently of the evaluated region (total, intratumoral, and peritumoral analysis; *P*<0.05; Fig. 2).

**Table 1:**
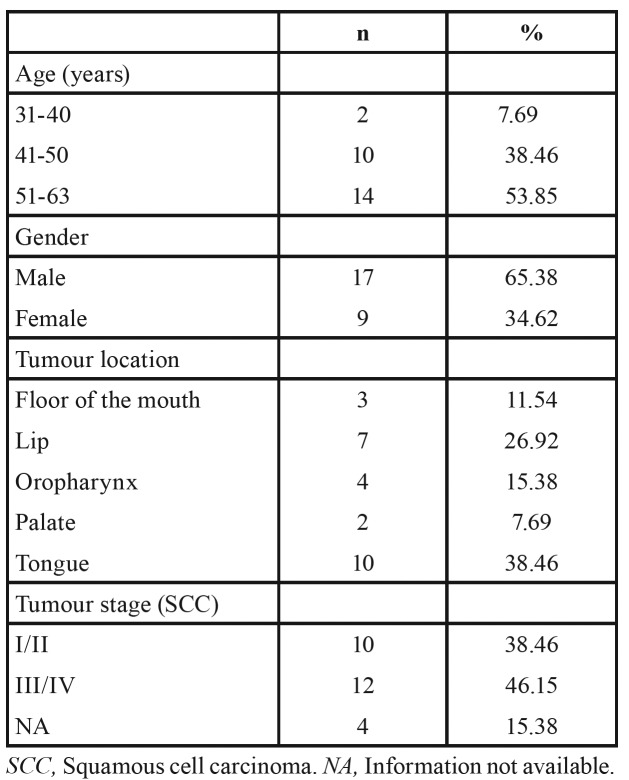
Distribution of patients according to age, gender, tumour location, and tumour stage.

The average positive cell counting values for all cases analysed in the intratumoral regions stained by CD1a were 57.66 for the SA method and 35.59 for the UA method; for CD83, the values were 8.25 cells (SA) and 5.54 cells (UA), and for CD207, the values were 25.41 (SA) and 16.70 (UA). Regarding the peritumoral regions, the average positive cell counting stained by CD1a were 83.40 (SA) and 19.38 (UA); for CD83, the values were 81.79 cells (SA) and 13.71 cells (UA); and for CD207, the average number of cells were 45.29 (SA) and 8.11 (UA).

The tumour location did not affect the positive DCs counting for any marker, independent of the quantification method (Table 2). Despite the statistics results, the lip located cases presented a higher density of positive DCs in the peritumoral regions, compared with the other locations (*P*>0.05). Different from the CD1a and CD207 markers, a higher number of CD83+ cells can be noticed in the peritumoral regions compared to the intratumoral regions. There was no positivity for CD83 in tumors located on the palate.

The tumour stage did not affect the positive DCs counting (*P*>0.05). However, a decreased of the DCs general values was observed according to the advance of the tumor stage (Table 3). Although no significant statistical differences were presented, some borderlines results are important to note. A decreasing of peritumoral CD1a+ cells were observed in advanced tumours stages (stage III/IV) (*P*=0.0901, SA method; *P*=0.0627, UA method). The same was observed for the peritumoral CD207+ cells; however, a relevant difference can be observed between the quantification methods *P* values (*P*=0.0583, SA method; *P*=0.7068, UA method).

Fifty-four percent of the patients (14 cases) had their SCCs classified histologically as well/moderately differentiated, whereas 46% (12 cases) were classified as poorly differentiated. The histological tumour grade affected only the intratumoral CD1a+ cells counting for both quantification method (*P*=0.0258, SA method; *P*=0.0340, UA method), and the peritumoral CD1a+ cells counting for UA method (*P*=0.0232; Table 4). For peritumoral CD1a+ cells, the number of positive DCs decreased in poorly differentiated tumours using the SA method, and the opposite happened with the use of the UA method. In general, the CD83 expression was more expressive in the peritumoral regions.

As expected, the positivity of DCs was higher in SCCs that presented prominent inflammation (Table 4). These results only were not significant for peritumoral CD83+ cells and intratumoral CD207+ cells. Regarding the quantification methods, differences were noted in 3 regions corresponding to the 3 different markers (intratumoral CD1a, intratumoral CD83, and peritumoral CD207; Table 4).

**Table 2:**
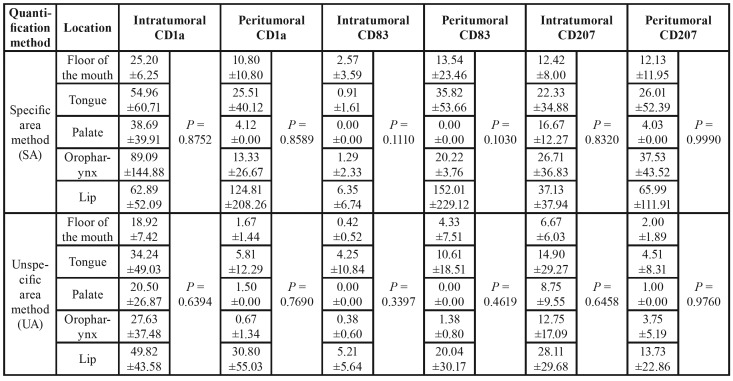
Mean ±standard deviation of positive cell counting according to the tumour location, quantification method (SA or UA) and the markers (CD1a, CD83, and CD207). *P* values obtained from ANOVA comparing the effect of tumour location (P-value<0.05; Student t-test).

**Table 3:**
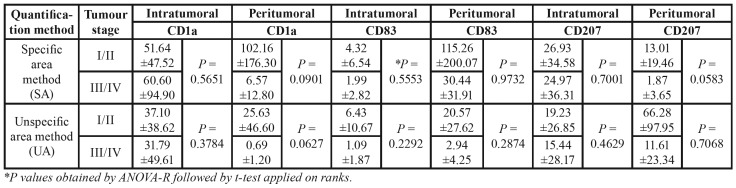
Mean ±standard deviation of positive cell counting according to the tumour stage (I/II and III/IV), the quantification method (SA or UA), and the markers (CD1a, CD83, and CD207). *P* values obtained from ANOVA comparing the effect of tumour stage (P-value<0.05; Student t-test).

**Table 4:**
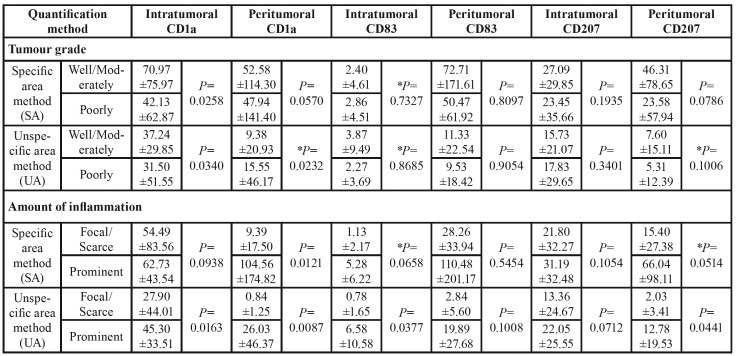
Mean ±standard deviation of positive cell counting according to the histological tumour grade and amount of inflammation, the quantification method (SA or UA), and the markers (CD1a, CD83, and CD207). *P* values obtained from ANOVA comparing the effect of histological grade and amount of inflammation (P-value<0.05; Student t-test).

## Discussion

This study focused on evaluate a novel DCs quantification method in SCC, and compare it with a conventional method. The findings showed that the positive cell quantification method influences the DCs density results and affected the DCs counting independently of the evaluated region. Unlike the conventional method (UA method), the novel SA method avoids non-target areas included in the hotspots improving the reliability and reproducibility of the cell quantification.

The cell quantification methods in the literature are heterogeneous in several aspects: the evaluation of different numbers of sequential/randomly fields (23,24,27-29) or the selection of positive cells hotspots (25); the use of different magnifications as counting areas (28,29) or the use of previously stipulated areas size (23,25); the use of the total number of cells (24,29) or the average of the evaluated fields (25). In addition, some studies that used manual methods reported the use of a reticulated square for the counting aid (23,27). Also, there are studies that did not clearly mention how the counting was performed (28,29). Despite the different available methods, the use of a stipulated number of hotspots and the average of the evaluated fields can reduce the influence of the tissue size variation present in the slides.

The use of digital quantification methods has been replacing the old manual methods, and they presumably would allow standardization of cell counting results. Therefore, the present study compared two different digital quantification methods (UA and SA methods) based on a technique variation and, evaluating the positive cell counting averages and *P* values presented in all Tables, it is possible to verify a difference between them. Furthermore, the present study showed contradictory results between the methods applied regarding the histologic tumour grade (Table 4). Herein, the SA method showed a decreasing positive DCs number in poorly differentiated tumours for peritumoral CD1a+ cells, and the UA method presented the opposite result. Corroborating the SA method findings, a previous study conducted by our research group (25) showed that decreased peritumoral CD1a+ cell number can predict a worse prognosis in oral SCC.

The difference between the quantification methods can be justified by the fact that the UA method does not consider the variability of morphological presentation of tumours or the different amounts of inflammation, so non-target areas can be incorrectly considered. The average of the demarcated areas (DA) for the SA method group analyses was 0.60 mm² ±0.23 mm² (mean ±standard deviation) for the intratumoral regions, and 0.21 mm² ±0.13 mm² for the peritumoral regions analyses. These results mean that, on average, around 40% of the intratumoral area and 80% of the peritumoral area corresponded to non-target areas that were considered on the 1mm² hotspots fields in the UA method. Thus, the present findings revealed that the quantification method influences the positive DCs density results, which leads to the acceptance of the research hypothesis.

Although the difference between the evaluated methods, corroborating previous findings (27), both methods showed a higher number of both mature and immature DC populations in the lip SCC samples than the other presented locations, which could contribute to establishing a more effective immune antitumor response for this neoplasm. In addition, the fact that lip cancer is related to different risk factors than oral and oropharyngeal cancer should be considered. In spite of the fact that 27% of our sample presented lip SCC, there is no information about the rise in lip cancer incidence in patients living with HIV. Also, even though it is known that different anatomical locations present different DCs number (30), the tumour location did not affect the positive DCs counting in the present study. This can be explained by the limited sample size effect.

Regarding the specific patient group evaluated in the present study, some authors (19,20) have suggested that HIV infection may be an independent factor, decreasing the density of LCs even in patients with normal CD4+ T cell counts and undetecTable viral load. In view of these findings, it is possible that the development of neoplasias and opportunistic infections in HIV-positive patients may be related to HIV infection even with the apparent immunological reestablishment achieved by the success of modern antiretroviral therapies. Also, the fact that HIV-positive patients with HNC seem to represents a different entity in terms of risk factors, prognosis, and treatment still needs to be elucidated. Thus, the next steps will correlate the present findings with the patients’ cART history and their CD4+ T cell counts. Also, an application of the same SA method in a control group (confirmed HIV-negative patients) and the collection of the recurrence rates and overall survival of the patients could lead to interesting results regarding the better understanding of the HIV-positive patients with HNC.

The limitations of the present study include the limited amount of cases retrieved and the restricted access to patients’ information due to the lack of standardized medical records and pathological reports. Also, regardless of the use of digital tools, a subjective aspect inherent to the methods always will be present, which reinforces the importance that analyses should be performed by an experienced pathologist. The main difficulty in the dendritic cell quantification is that the dendrites can represent single or various cells, depending on the tissue structure and slice width. Thus, despite the amount and diversity of studies present in the literature, there is no gold-assay regarding the positive cell quantification system. However, the authors believe that following the methodology presented and discussed above, the reliability and reproducibility of the DCs quantification will significantly improve.

There is a heterogeneity of the quantification methods presented in the literature, including the studies that evaluate the density of DCs (19,22-25,27-29). Since we have shown that the quantification method can affect the research results and may lead to the acceptance or not of their significance, the necessity of a cell counting methodology with higher reliability is enforced. Hence, we encourage the use of the presented method idea (SA method) for any positive cell type counting, which also could facilitate the use of results as a reference for future studies.
